# Conformity assessment of a computer vision-based posture analysis system for the screening of postural deformation

**DOI:** 10.1186/s12891-022-05742-7

**Published:** 2022-08-22

**Authors:** Kwang Hyeon Kim, Moon-Jun Sohn, Chun Gun Park

**Affiliations:** 1 Department of Neurosurgery, Neuroscience and Radiosurgery Hybrid Research Center, Inje University Ilsan Paik Hospital, College of Medicine, 170 Juhwa-ro Ilsanseo-gu, Gyeonggi province 10380 Goyang, South Korea; 2 Department of Mathematics, Kyonggi University, Gwanggyosan-ro, Yeongtong-gu, 16227, Suwon, South Korea

**Keywords:** Computer vision, Postural spinal deformity, Scoliosis, Clinical decision support system, Principal component analysis

## Abstract

**Background:**

This study evaluates the conformity of using a computer vision-based posture analysis system as a screening assessment for postural deformity detection in the spine that is easily applicable to clinical practice.

**Methods:**

One hundred forty participants were enrolled for screening of the postural deformation. Factors that determine the presence or absence of spinal deformation, such as shoulder height difference (SHD), pelvic height difference (PHD), and leg length mismatch (LLD), were used as parameters for the clinical decision support system (CDSS) using a commercial computer vision-based posture analysis system. For conformity analysis, the probability of postural deformation provided by CDSS, the Cobb angle, the PHD, and the SHD was compared and analyzed between the system and radiographic parameters. A principal component analysis (PCA) of the CDSS and correlation analysis were conducted.

**Results:**

The Cobb angles of the 140 participants ranged from 0° to 61°, with an average of 6.16° ± 8.50°. The postural deformation of CDSS showed 94% conformity correlated with radiographic assessment. The conformity assessment results were more accurate in the participants of postural deformation with normal (0–9°) and mild (10–25°) ranges of scoliosis. The referenced SHD and the SHD of the CDSS showed statistical significance (*p* < 0.001) on a paired t-test. SHD and PHD for PCA were the predominant factors (PC1 SHD for 79.97%, PC2 PHD for 19.86%).

**Conclusion:**

The CDSS showed 94% conformity for the screening of postural spinal deformity. The main factors determining diagnostic suitability were two main variables: SHD and PHD. In conclusion, a computer vision-based posture analysis system can be utilized as a safe, efficient, and convenient CDSS for early diagnosis of spinal posture deformation, including scoliosis.

## Background

A clinical decision support system (CDSS) is driving the paradigm shift in healthcare [1]. The CDSS is defined as a computer system designed to assist clinicians in helping decisions for individual patients in the healthcare or medicine fields [2]. The advantage of CDSS is to reduce unnecessary screening of patients and ultimately ensure patient safety by providing accurate diagnostic results to clinicians [1]. CDSS has recently been used not only in the field of diagnosis using medical images and data, but also in the field of diagnosis and outcome prediction in combination with computer vision systems, artificial intelligence algorithms, and advanced analysis software functions [3–5].Meanwhile, posture deformation is one of the changes led by spinal deformity, and scoliosis is the lateral curvature of the spine [6]. Adolescent idiopathic scoliosis (AIS) is the most common type of scoliosis [7]. It can be diagnosed when a radiographic Cobb angle is greater than 10°. AIS affects 1 to 4% of adolescents during early puberty and is more common in young women than in young men [8]. Early diagnosis is often difficult because there are no symptoms such as pain, and missing the optimal time to improve with conservative treatment leads to structural spinal deformation, which adversely affects spinal health. For example, exercise can improve a patient's quality of life by reducing the progression of spinal curvature [9]. It has been reported that most mild scoliosis and spinal deformity including nonstructural or functional scoliosis except for structural scoliosis can be improved by posture correction and exercise [10–13]. A simple screening method with non-radiographic analysis for diagnosing AIS is being used and studied due to radiation exposure although radiographic diagnosis is basically used for detecting scoliosis [14–17]. In addition, the demand for simple scoliosis screening methods is expected to detect the postural deformity according to the increasing aging spine [18–20]. Although simple radiographic imaging tests have traditionally been used to diagnose postural deformity, including idiopathic scoliosis, the use of non-ionizing radiation has been recognized as a limitation in adolescence. To alleviate diagnostic x-ray hazard, direct body measurement and the Moiré pattern method in the coronal plane was developed and used clinically for the early diagnosis of postural spinal deformity [21, 22]. In most participants with relatively low angle deformation (< 25°) of the spine, CDSSs without non-ionized radiation diagnostic devices have emerged as appropriate screening diagnostic tools in the coronal plane of the spine. CDSS studies have been reported for early detection of AIS. Recently, several studies for scoliosis screening have used deep learning and machine learning to predict curve progression and curvature classification that using comparative images of scoliosis and normal spine curvature with a training dataset [23, 24]. In the meantime, the difference in the height of major joints in the body is an indicator of the state of spinal deformity [25]. Thus, it is necessary to evaluate the postural deformation of the evaluation of the numerical values by calculating the difference in the height of each joint based on the body posture. In this study, a computer vision-based posture analysis system such as the CDSS that uses non-ionizing radiation was used to evaluate the accuracy of screening for detecting postural deformity. The early screening for the detection of postural spinal deformity is the starting point of the scoliosis diagnosis using an in-depth imaging method. Therefore, the parameters used to detect the postural deformation were analyzed through conformity assessment and principal component analysis.

### Materials and methods

#### Participants and CDSS posture analysis system

**Fig. 1:**
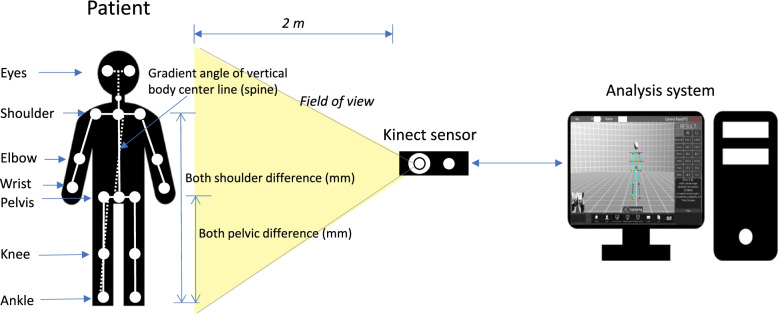
Examination diagram of the postural analysis system. An image of the skeleton of the participant in the standing position is acquired via a kinetic camera. The length and angle differences for the main points on each body part (centerline of the body, shoulder, pelvis, and ankle) are analyzed

One hundred forty participants were enrolled to evaluate the postural deformation in our institution from Jun 2017 to Jun 2018 (*n* = 140). The inclusion criteria were the participants who came to the hospital to diagnose the functionality of postural imbalance, non structural postural deformity, or the presence of scoliosis (including adolescent idiopathic scoliosis) through a screening test. In contrast, the exclusion criteria in the present investigation were as follows: (a) patients with diseases requiring surgical treatment on medical imaging examination (L spine MRI or Pelvic MRI), (b) patients with pyogenic and other inflammatory diseases, (c) congenital patients with spinal deformity, (d) structural scoliosis patients, (e) patients with secondary pain due to dystonia or a causal disease not related to contraction or spasm, (f) patients with moderate and major double curve type, and (g) participants who have had surgery for diseases of the musculoskeletal system (spine, pelvis, hip joint, knee, ankle, and flat foot). The study has obtained the institutional review board approval (IRB No. 2022–03-016–001). The CDSS (PA3017; Driom, Incheon, South Korea) is a computer equipped with a kinect sensor and analysis software (Fig. 1). Participants stand in front of the kinect sensor at a distance of approximately 2 m, and an image is captured on the computer. The participant looks in front of the camera and walks in place for about 10 steps while moving their arms. In this process, each joint of the body is recognized for movement by computer vision (Fig. 1). After that, the participant remains in a comfortable position and the computer vision analysis is completed. The software then analyzes the skeleton. A moving image is also recorded to allow the computer to distinguish the edges of the participant's body and joints. Once the joints are identified by the software, it can determine the skeletal structure and the gait of the participant. The program's algorithm for judging scoliosis sets the central coronal axis of the body using a connecting line through the participant's eyes, shoulders, and pelvis. Also, the gradient angle of the vertical body centerline is determined in the CDSS. The probability of scoliosis is determined using the angle of curvature of this axis (normal range ≤ 3 mm; 20% scoliosis = 3–10 mm; 50% scoliosis ≥ 10 mm) by the algorithm of the CDSS. For each CDSS, the range of grouping that classifies the severity using the cobb angle may be different [26]. In this study, normal (0–9°), mild (10–25°), moderate (26–40°), and severe (> 41°) categories were used to group the diagnostic range of the Cobb angle.

#### Radiographic parameter analysis

For radiographic postural deformation analysis, an X-ray image was obtained of each participant in a standing position. On the measured X-ray images, pelvic height difference (PHD), shoulder height difference (SHD), and Cobb angle were analyzed for each participant using length and angle measurement tools (M6, Infinity Ltd., Seoul, South Korea). Participants’ scoliosis were analyzed by Cobb angle division by normal (0–9°), mild (10–25°), moderate (26–40°), and severe (> 41°) range in degrees [27]. A paired t-test was performed to determine the diagnostic relationship between parameters from CDSS and the radiographic assessment (*p* < 0.05 significance level). The data were obtained by radiographic analysis and postural deformation diagnosis support system.

#### Conformity evaluation of the posture analysis system

To evaluate the postural deformation screening support system, the results of a radiographic postural deformation analysis were used as a reference. In the radiological evaluation and CDSS analysis, conformity is evaluated as to whether the result is the same in 4 stages ranging from normal to severe group. The range of scoliosis probability (%) calculated from CDSS is divided into 0, 20 to 50, 50 to 65, and > 65% or higher. CDSS outputs the result score of posture deformation divided into 4 score groups for screening suspected scoliosis. And the level difference with the radiographic assessment range (normal, mild, moderate, and severe range) corresponding to the CDSS results was compared. For example, if the Cobb angle from the radiographic assessment is 5° and the result from CDSS is 0°, the conformity is a 100% score. However, if the result from CDSS predicted 25%, the conformity would have 75% for a difference of 2 levels.

Based on this, the conformity of the postural deformation screening system was schematized for the individual diagnostic results of the 140 participants. The conformity is simply defined as the ratio of *SS* and *RS* and is expressed as Eq. (1).

$$conformity=\frac{SS}{RS} (1)$$

where *SS* is the score of the postural deformation for screening suspected scoliosis from the CDSS, and *RS* is the reference score from the radiographic postural deformation evaluation.

#### Principal component analysis for CDSS

The indices we obtained from the CDSS were a total of 9 parameters including SHD and PHD in Table 1. The principal component analysis is one of the most well-known methods for extracting the most important factors. That is, the importance between variables is divided into the first principal component, and the second principal component. In other words, the main factors or components that had the greatest influence on a certain analysis result are obtained among several parameters. Here's a more detailed explanation of this: Principal component analysis (PCA) can be thought of as fitting a p-dimensional ellipsoid to the data [28]. Here each axis of the ellipsoid represents a principal component (PC). To find the axis of the ellipsoid, we first subtract the mean of each variable from the dataset so that the data are centered around the origin. We then compute the covariance matrix of the data and compute the eigenvalues and corresponding eigenvectors of this covariance matrix. Then, we normalize each orthogonal eigenvector to convert it to a unit vector. Once this is done, each mutually orthogonal unit eigenvector can be interpreted as an axis of an ellipsoid that fits the data. This criterion selection transforms the covariance matrix into a diagonal form with the diagonal elements representing the variance of each axis. The ratio of variance represented by each eigenvector can be calculated by dividing the eigenvalue corresponding to the eigenvector by the sum of all eigenvalues. In other words, an element that matches or has a direction to concerning the direction of this vector can be interpreted as a correlated variable [29]. The software used for analysis comprised Python 3.8.3, Scikit-learn 0.23.1, SciPy 1.5.0, and Stats Models 0.11.1 [29].

**Table 1:** Participants’ characteristics from the computer vision-based posture analysis system (*n* = 140)

Category	Characteristics		Mean ± SD or No. (%) or IQR
**Parameters from computer vision-based posture analysis system**	Age (years)		24.94 ± 17.36
	Sex	Male	59 (42.14%)
		Female	81 (57.86%)
	Height (cm)		153.43 ± 18.57
	Weight (kg)		51.51 ± 18.13
	SHD (mm)		3.00 ± 1.00
	EHD (mm)		8.50 ± 0.50
	WHD (mm)		39.00 ± 19.00
	PHD (mm)		7.50 ± 2.50
	KHD (mm)		10.00 ± 2.00
	AHD (mm)		18.50 ± 4.50
	LLD (mm)		7.67 ± 5.27
**Radiographic assessment**	Cobb angle	Including 0 (*n* = 140)	6.16, IQR Q1: 0.00, Q3: 10.25 (0.00–61.00)
		Excluding 0 (*n* = 79)	10.92, IQR Q1: 6.00, Q3: 13.00 (1.00–61.00)
	SHD (mm)	Including 0 (*n* = 140)	1.18, IQR Q1: 0.00, Q3: 0.00 (0.00–18.20)
		Excluding 0 (*n* = 20)	8.25, IQR Q1: 3.98, Q3: 10.73 (1.30–18.20)
	PHD (mm)	Including 0 (*n* = 140)	2.85, IQR Q1: 0.00, Q3: 5.10 (0.00–18.60)
		Excluding 0 (*n* = 58)	8.04, IQR Q1: 4.15, Q3: 8.43 (1.80–18.60)
	Scoliosis range in degree (Cobb angle)	Normal group (0–9°)	96 (68.57%)
		Mild group (10–25°)	41 (29.29%)
		Moderate group (26–40°)	1 (0.71%)
		Severe group (> 41°)	2 (1.43%)

*Abbreviations*: *SHD* Shoulder height difference, *EHD* Elbow height difference, *WHD* Wrist height difference, *PHD* Pelvic height difference, *KHD* Knee height difference, *AHD* Ankle height difference, *LLD* Leg length discrepancy, *IQR* Interquartile range

## Results

**Fig. 2:**
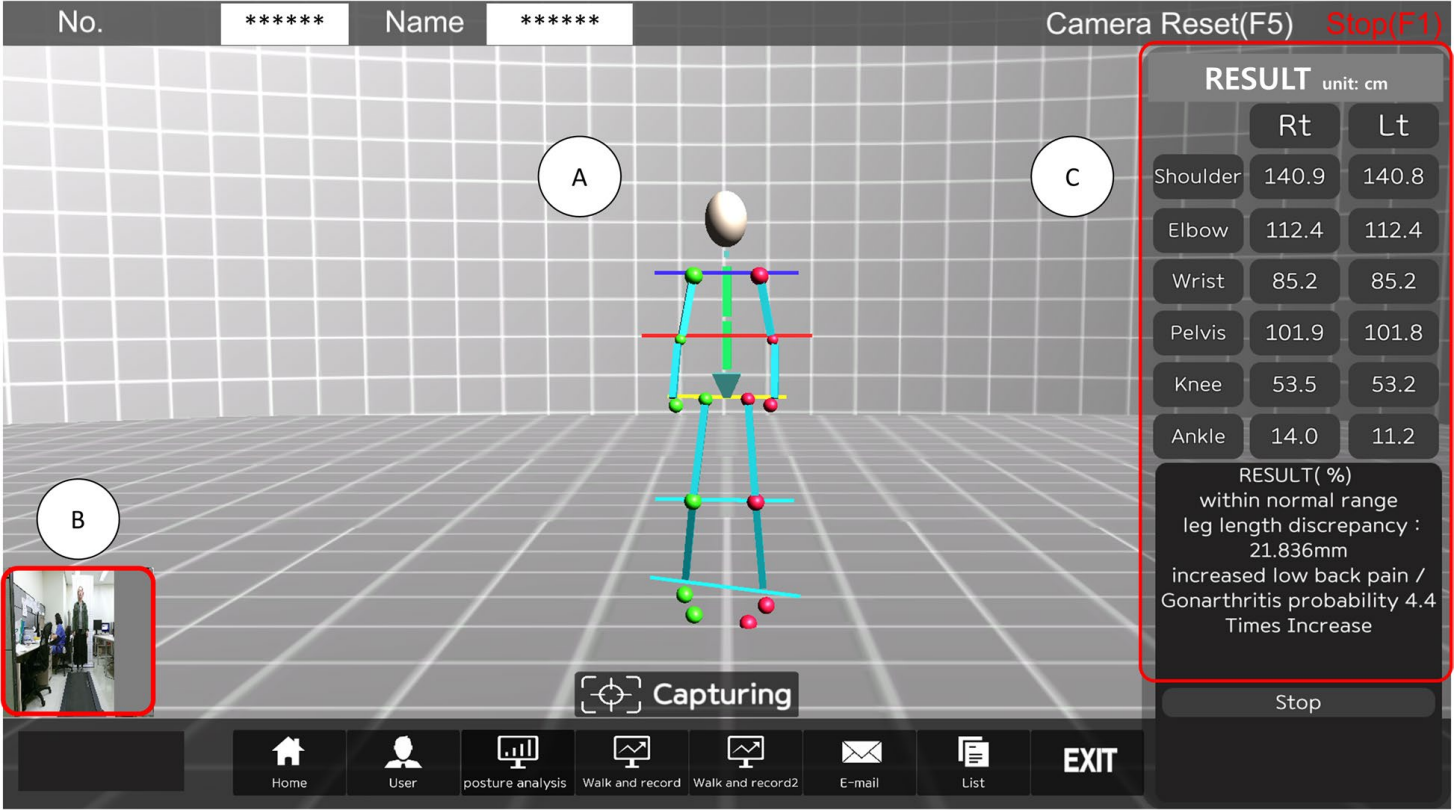
The user interface of the diagnosis system for screening postural deformation. The user interface of the postural deformation diagnosis system during the examination of a participant. a Image of the skeletal structure of a participant in a standing posture. b A participant in front of the kinetic camera. c Decision support analysis result for a participant based on shoulder, pelvis, and leg length information

The CDSS was used in screening 140 participants with postural deformation (Fig. 2). When the participant stands in front of the camera and moves the joints of the body, the screen is shown in Fig. 2a is displayed. A real-time image of a participant during the examination is shown in Fig. 2b. The lengths between the points representing each body part are measured in the standing posture, and the diagnosis is displayed based on these measurements (Fig. 2c). 140 participants were analyzed individually (Table 1). Participant body points were analyzed for each body part in Fig. 2. For most body parts, the difference could be analyzed with an average of about 3 mm or more through the computer vision-based posture analysis system in Table 1. Standing posture X-rays were obtained from the 140 participants. Through the radiographic assessment, 75% of participants were diagnosed with the normal range for scoliosis, 22.86% of participants were diagnosed with the mild range of scoliosis, and 0.71% and 1.43% of participants were diagnosed with a moderate and severe range of scoliosis, respectively. The PHDs and SHDs were analyzed using X-ray images, and the Cobb angles were calculated. The Cobb angles of the 140 subjects ranged from min 0° to max 61°. The mean was 6.16 ± 8.50°. Statistical analysis results using paired t-test for the major parameters between the clinical decision support system and the referenced radiographic analysis were shown in Table 2. The shoulder height difference and referenced radiographic postural deformation results analyzed through the CDSS were compared with a paired t-test (Table 2). There was a statistical significance between the CDSS shoulder height difference (SHD) and the referenced SHD (*p* < 0.001). The CDSS calculated the gradient angle of the vertical body centerline to calculate the scoliosis probability as in Fig. 1. It was compared with the Cobb angle in terms of the variable determining the probability of spinal deformity. The gradient angle for the postural deformation result and the Cobb angle analysis result was also analyzed and a statistical difference was found (*p* < 0.001) (Table 2).

**Table 2:** Statistical analysis results using paired t-test for the major parameters between the clinical decision support system and the referenced radiographic analysis (*n* = 140)

Parameters	CDSS results	Radiographic assessment	*p*-value
**Difference**	SHD (mm)	SHD (mm)	< 0.001
	PHD (mm)	PHD (mm)	0.513
	LLD (mm)	LLD (mm)	0.053
**Outcome**	Gradient angle (°)	Cobb angle (°)	< 0.001

Notation: SHD, shoulder height difference; PHD, pelvic height difference; LLD, leg length discrepancy; Gradient angle of the vertical body for determination of patient’s scoliosis probability

### Parameter analysis using correlation and PCA

**Fig. 3:**
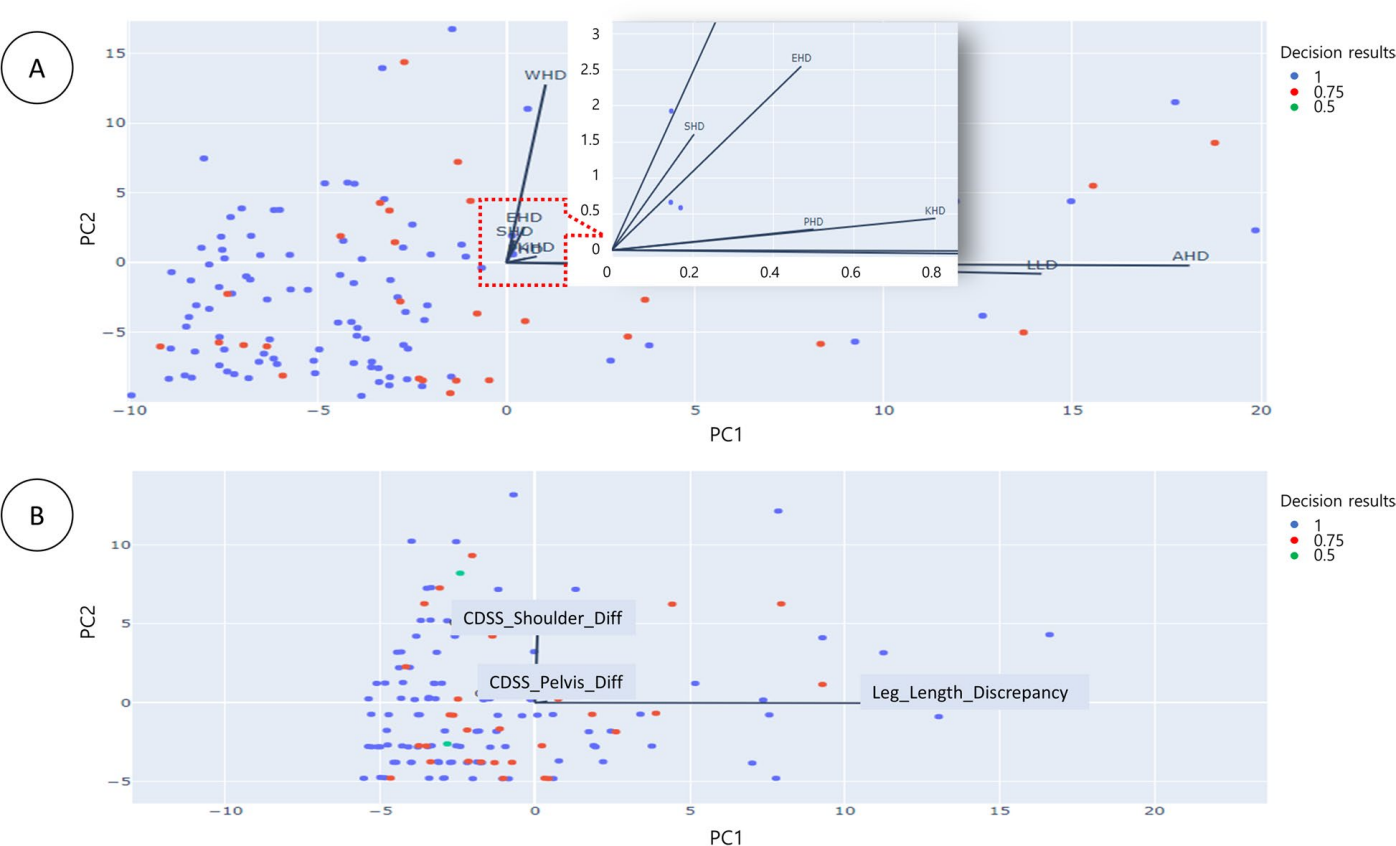
Principal component analysis for the decision results. Principal component analysis for the decision results for the participants (*n* = 140). a Eigenvectors of the parameters extracted from CDSS with principal components axes (**b**) Main parameters for SHD, PHD, and LLD as principal components. (Abbreviations: PC1, Principal component 1; PC2, Principal component 2; SHD, Shoulder height difference; EHD, Elbow height difference; WHD, Wrist height difference; PHD, Pelvic height difference; KHD, Knee height difference; AHD, Ankle height difference; LLD, Leg length discrepancy; CDSS, Clinical decision support system; Diff, Difference)

**Fig. 4:**
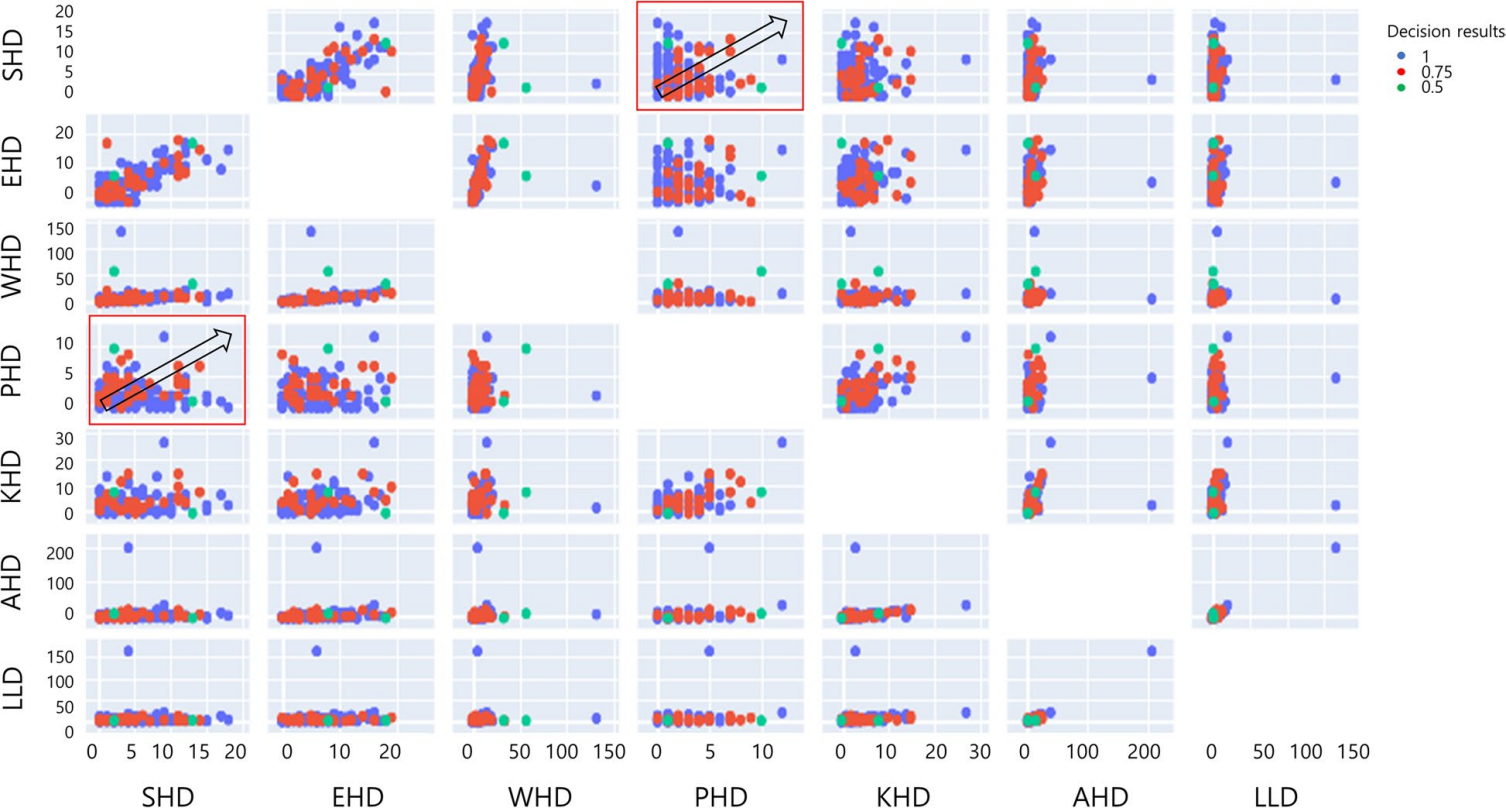
Principal component analysis in detail for the parameters in important order. Principal component analysis for the decision results for the participants (*n* = 140). (Abbreviations: SHD, Shoulder height difference; EHD, Elbow height difference; WHD, Wrist height difference; PHD, Pelvic height difference; KHD, Knee height difference; AHD, Ankle height difference; LLD, Leg length discrepancy)

**Fig. 5:**
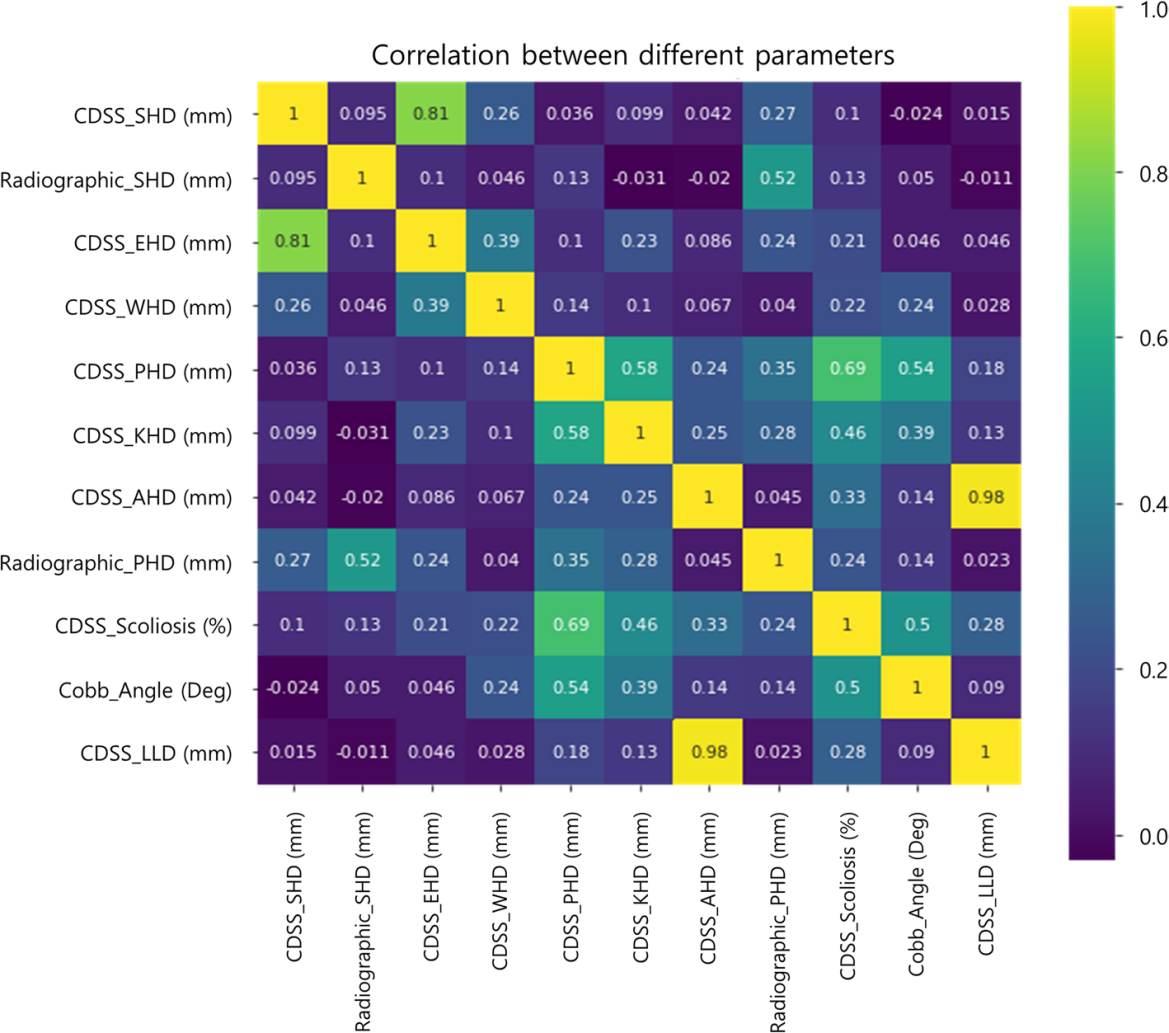
Correlation analysis for the parameters. Correlations between the clinical decision support system and the referenced radiographic analysis for the parameters used to diagnose postural deformation

First, a PCA was performed on the decision support results of postural deformation obtained using the CDSS (Fig. 3 and 4). Figure 3a shows the analysis of principal components (PC) for the postural deformation diagnosis using radiographic analysis. The SHD and the elbow height difference (EHD) components were analyzed as PC for the principal component 1 (PC1) and principal component 2 (PC2) axes, respectively. This is shown as a vector (PC1 = 34.40%: SHD, PC2 = 27.13%: EHD). However, Fig. 3b shows a schematic diagram of mainly dominant parameters for the SHD, PHD, and leg length discrepancy (LLD) on the CDSS. The EHD and wrist height difference (WHD) are in the same direction (eigenvectors) for the SHD and knee height difference (KHD) and ankle height difference (AHD) are the same as PHD (Fig. 3a and 4). SHD and LLD are orthogonal in the eigenvector direction (Fig. 3b). Thus, SHD and PHDs as PC were the predominant factors (PC1 = 79.97%: SHD, PC2 = 19.86%: PHD and PC3 = 0.17%: LLD). Second, the correlations between the major factors analyzed through the CDSS and the major factors analyzed through a referenced radiographic analysis were analyzed (Fig. 5). The SHD for the CDSS and the EHD for the CDSS were relatively highly correlated (81%) in Fig. 4. Also, the AHD for the CDSS and the LLD for the CDSS were relatively highly correlated (98%). The PHD and scoliosis results (69%) obtained using the CDSS were relatively highly correlated for each factor. CDSS made a more accurate diagnosis of scoliosis in participants with a large pelvic height difference than a small shoulder height difference.

### Accuracy evaluation of the CDSS

**Fig. 6:**
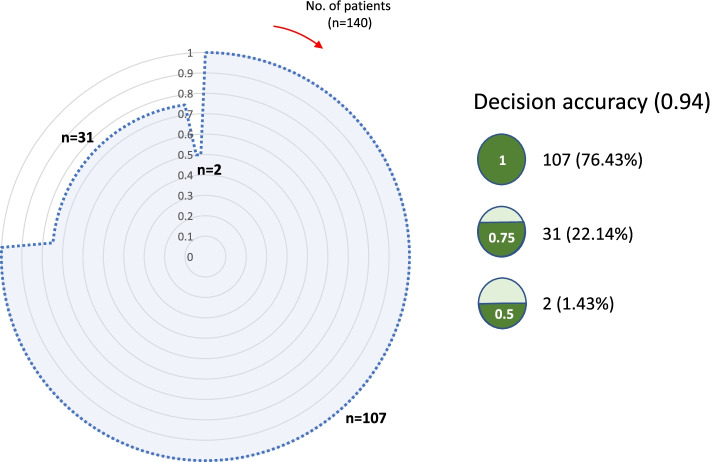
Total conformity circle for individual decision results for scoliosis (*n* = 140)

For the individual participants, the analysis result on the CDSS was consistent with the result found by referenced radiographic analysis (*n* = 107, 76.43%). In addition, 31 of the participant’s outcomes showed a one-level (Cobb angle 10–25°) difference between referenced scoliosis results and CDSS results (*n* = 31, 22.14%). In addition, there was a difference of two or more levels (more than 25–40°) between CDSS results and referenced results for two participants (*n* = 2, 1.43%) (Fig. 6). In Fig. 6, the area of the outer circle (the largest circle marked with a solid line which is the referenced accuracy) is 1. The diagnostic accuracy of the scoliosis diagnosis system is shown in light dotted blue. And the accuracy is 0.94.

## Discussion

Since moiré topography was used for scoliosis screening in the 1970s [21, 22], 3D surface topography in the 2000s [30], recently the computer vision-based CDSS [14, 17], and AI-based CDSS have evolved [23, 24, 31–36]. However, there is a pitfall of CDSS in addition to the benefits mentioned earlier introduction section. CDSS mainly depends a lot on the computer performance and hardware specification [1]. The performance of the algorithm for specific decision support should be validated so that clinicians can accept this result. The studies using CDSS for scoliosis screening using non-radiographical methods are summarized in Table 3. They assessed the accuracy of each CDSS with the validation methods. For example, Yıldırım et al. [17] used 3D topography from a 3D scanner for the diagnosis of adolescent idiopathic scoliosis. Overlapping the diagnostic system and medical images using a hand-held scanner, calculating the root mean square in mm units through point-to-point measurement, and correlation with the Cobb angle (r_max_ = 0.92, r_min =_ 0.47 in standing posture) have also been analyzed as diagnostic methods. Lai et al. [37] compared the angle differences (2.9° ± 1.8°) found with a conventional 3D ultrasound imaging system with those obtained on a portable 3D ultrasound imaging system. In our study, referenced analysis was performed using X-ray radiographs to evaluate the parameters (Table 1) including the Cobb angle, which is regarded as a useful standard in Eq. (1). That is, this study is evaluated as a topic for solving the shortcoming of using ionizing radiation for screening and analysis by evaluating the screening results from a computer-based CDSS through the conformity assessment. In addition, the dataset associated with parameters extracted from CDSS will be the training datasets of the artificial intelligence or additional data analysis in the next study.

**Table 3:** Evaluative studies of the computer vision-based analysis system for scoliosis screening

Author	Patients (n)	Computer vision	Comparison/evaluation	Results	Year
**Yıldırım et al. **[17]	42	Hand-held 3D scanner with tablet	Conventional ultrasound scoliosis diagnosis system/point-to-point matching, correlational analysis	Root mean square, correlation (r_max_ = 0.92, r_min =_ 0.47 in standing posture)	2021
**Lai et al. **[37]	19	3D ultrasound imaging system	Commercial 3D ultrasound imaging system/absolute dataset difference	Absolute difference between the two data sets (2.9° ± 1.8°)	2021
**Zhang et al. **[34]	367	Built-in smartphone camera	Plain X-ray images/deep learning-based vertebral landmark detection and difference analysis	Average L2 error (2.8 pixels), Recall (0.99)	2021
**Cho et al. **[36]	629	U-net segmentation, binary mask	Plain X-ray images/Cobb angle measurement difference	Matching score (0.821), mean absolute error of 8.055° for Cobb angle	2020
**Mishra et al. **[33]	22	3D vision with surface topography	Plain X-ray images/topographical differences	Standard deviation (± 3.4)	2020

### Feature extraction of the parameters related to postural spinal deformity

PCA is a technique for finding the axis of the principal component by transforming samples in a high-dimensional space into a low-dimensional space while preserving the variance of the data [28]. In the process of merging these highly related features into one, the PCA results were derived. The features could be sorted by two main variables related to SHD and PHD in Fig. 3b. That is, the same direction of the eigenvectors of WHD, EHD, and SHD are closely related to each other in Fig. 3a. On the other hand, PHD is related to AHD, KHD, and LLD. And the orientation of each eigenvector of the parameters may result in the variation approaching 0 or increasing widely in Fig. 4 to screen for postural deformation. Thus, an intuitive interpretation of the PCA is possible for this study.

### Conformity of this CDSS system through the cross-evaluation

This system showed 94% accuracy for postural deformation. It means conformity was highly guaranteed by our accuracy evaluation (Fig. 6). Radiographic diagnoses were categorized as normal level of scoliosis (68.57%, *n* = 96) and mild to severe range of scoliosis (31.43%, *n* = 44) for 140 individual participants. The participants with a normal level of scoliosis were included inaccurate decision result for conformity assessment in Fig. 6. If more increased participants of the moderate and severe levels of scoliosis are included in the overall dataset, the conformity result may be different. In the referenced Cobb angle analysis of the 140 participants, the angle ranged from 0° to 61°, with a mean of 6.16 ± 8.50°. Therefore, it is desirable to utilize the CDSS with the goal of convenient use for the diagnosis of normal and low-level scoliosis to relatively high-level scoliosis. The main function of postural deformation diagnosis is to provide evidence that more precise X-ray, MRI, and CT examinations are needed to select patients with severe structural deformities who require surgery. Thus, it was confirmed that safe postural deformation screening with non-ionizing radiation is possible by utilizing the analysis system.

### Limitations of spinal deformation analysis for participants with double curved and kyphosis

The curve types of the participants analyzed in this study were lumbar, thoracolumbar, and thoracic curve types. As a result of the radiographic assessment of 140 participants, almost participants (> 99%) had a single curve or a few mild double curve participants less than 2–3°. Thus, the profile of participants with moderate and major double curve types was not included in the analysis. The CDSS is likely to output high scores in spinal deformity probability based on parameters such as SHD and PHD, but further studies on analytical accuracy measurement are needed. Some patients are often accompanied by thoracic kyphosis rather than scoliosis. Therefore, lateral radiographic images are required to observe sagittal spinal deformity. In this study, the spinal deformity was evaluated from the coronal plane by using a frontal view kinetic-image sensor. Thus, hyperkyphosis could not be evaluated from the sagittal view. In the future study, a lateral imaging view system can be considered for overcoming this limitation. The CDSS determines spinal deformity by comprehensively considering the gradient angle of vertical body centerline, SHD, and PHD. It is difficult to compare these parameters with radiographic assessment exactly one to one like the variable of Cobb angle. However, there is an aspect that can be compared with the most similar parameters such as PHD and SHD.

## Conclusion

The CDSS showed 94% conformity for the screening of postural deformation. And the principal components could be sorted by two main variables related to SHD and PHD. Although most of the patients analyzed in this study had minor postural deformity, the computer vision-based posture analysis system using non-ionizing radiation is an efficient and clinically convenient screening and diagnostic tool for suspected scoliosis.

## Data Availability

Not applicable.

## Abbreviations

CDSS: Clinical decision support system
AIS: Adolescent idiopathic scoliosis
PHD: Pelvic height difference
SHD: Shoulder height difference
SS: Score of the postural deformation for screening suspected scoliosis from the CDSS
RS: Reference score from the radiographic postural deformation evaluation
PCA: Principal component analysis
PC: Principal component
PC1: Principal component 1
PC2: Principal component 2
SHD: Shoulder height difference
EHD: Elbow height difference
WHD: Wrist height difference
KHD: Knee height difference
AHD: Ankle height difference
LLD: Leg length discrepancy
